# The Progress of Gout Prediction Models Based on Multi-source Data

**DOI:** 10.2174/0115733971444947260203111252

**Published:** 2026-03-30

**Authors:** Wenrui Shi, Hongzhu Qu, Xiangdong Fang

**Affiliations:** 1 Department of Application Development, China National Center for Bioinformation, Beijing, 100101, China;; 2 Application Development Department, Beijing Institute of Genomics, Chinese Academy of Sciences, Beijing, 100101, China;; 3 College of Life Sciences, University of Chinese Academy of Sciences, Beijing, 100049, China

**Keywords:** Gout, urate, multi-omics, polygenic risk score, predictive model, diagnostic model

## Abstract

**Introduction:**

Gout, a highly serious inflammatory disease that is caused by monosodium urate crystals, is becoming an increasingly significant health concern. Artificial Intelligence and multi-omics-based research have made significant gains for the early detection and prevention of gout based on diverse approaches. This review intends to summarize current advances in forecasting gout susceptibility and gout-related symptoms, evaluate the predictive efficacy of different features, and ascertain which clinical and omics characteristics are most effective in these prediction models.

**Methods:**

We explored the PubMed database after 2010 using keywords such as “gout”, “predictive model”, “risk prediction”, and “machine learning”, and confined our search to English-language articles. The original peer-reviewed research articles that developed gout models were selected. Research that was not original or lacked internal validation was excluded.

**Results:**

Clinical features, genomics, microbiomics, radiomics, and metabolomics have been utilized to construct models related to gout and have demonstrated excellent predictive performance. Multisource data prediction models usually exhibit better effectiveness.

**Discussion:**

Gout-oriented models performed excellently in predictive performance but present limitations in certain clinical and omics domains. However, if they are to affect actual patient care, they must overcome some external confirmation roadblocks and the fiscal and practical implications they will face ahead of time.

**Conclusion:**

This review indicates that clinical and multi-omics models of gout are significant instruments for clinical decision-making. The models constructed in these studies may be crucial for the treatment of gout and its practical benefits.

## 1 INTRODUCTION

Gout is a metabolic arthritis stemming from impaired purine metabolism, marked by intense pain and limited joint motion, with a strong tendency to recur [1, 2]. The worldwide occurrence of gout spans from 0.1% to 10%, showing a rising yearly incidence [3, 4]. Hyperuricemia, caused by increased blood uric acid levels (>7 mg/dL), is a vital risk factor in gout's development. Elevated blood uric acid concentrations result in the deposition of monosodium urate crystals in joints and surrounding tissues and the release of various pro-inflammatory cytokines, such as interleukin-6, thereby triggering acute gouty arthritis [5, 6]. Health studies indicate that around 10% of hyperuricemia sufferers will ultimately experience typical gout symptoms during the illness's advancement [7, 8]. Gout will bring considerable medical and financial strains upon individuals while endangering their sustained quality of life and health [9].

Prompt prevention of gout can reduce its incidence. This permits clinicians to quickly formulate focused intervention plans, which include lifestyle alterations, dietary adjustments, and initiating uric acid-lowering treatment (ULT) therapy^.^[10, 11]. Given the advancement of big data and Machine Learning (ML) methods, investigators have built high-utility prediction and detection models for gout, capable of precisely measuring various hazards before and after gout onset. These models provide scientific evidence and decision-making support for the early prevention and course management of gout [12, 13]. We found that 74% of the final available studies focused on predicting gout onset, while 26% focused on forecasting post-onset clinical presentations (Table **1**). With advances in multi-omics techniques, new studies using metabolomic, microbiomic, and radiomic profiles have provided additional vital attributes for predicting gout. These multi-omics datasets allow thorough risk grouping from supplementary biological perspectives, thereby boosting model accuracy and practical value. This development is very important and promising to enhance early diagnosis, risk prediction, and the formulation of focused strategies for reducing gout [14].

## 2 REVIEW METHODOLOGY

### 2.1 Search Strategy

A systematic search of the PubMed databases for literature after 2010 using the following keywords and MeSH terms: “gout,” “predictive model,” “risk prediction,” “machine learning,” *etc*. The retrieval strategy was built for the database and used studies based on gout prediction models (917 publications), literature in the English language.

### 2.2 Search Results

The literature search identified a wide array of publications connected to gout modeling investigations. Selected studies were peer-reviewed original research that developed gout models, while non-original studies and those missing internal verification were excluded (excluding 785 publications). Consistent with the narrative review method, the selection emphasized studies offering valuable perspectives on gout model development and confirmation.

## 3 GOUT PREDICTION MODELS WITH SINGLE-SOURCE FEATURES

### 3.1 Gout Prediction Models Based on Clinical Features

Clinical features serve as disease-specific indicators that directly reflect pathological abnormalities in patients. Characterized by easy accessibility, low cost, and strong clinical interpretability, clinical features constitute fundamental indicators for disease prediction models and play a critical role in gout risk assessment [15, 16] (Table **2**).

Traditional statistical models based on clinical features remain indispensable in disease risk prediction due to their strong interpretability, statistical causal relationships with the disease, lower sample size requirements, and close alignment with clinical guidelines. A multivariate logistic regression model, constructed using 8 serum biochemical markers, showed high accuracy in predicting different clinical manifestation phases of gout, with the overall receiver operating characteristic curve (ROC-AUC) of 0.9534, demonstrating the clinical utility of traditional statistical models for gout risk prediction [17].

With the advancement of artificial intelligence technology, ML prediction models, capable of handling non-linear complex relationships, efficiently processing multi-source heterogeneous data, and possessing strong generalization ability and prediction accuracy, are playing an increasingly critical role in disease risk prediction [18-20]. The ML models have demonstrated a strong ability to predict the risk of gout in Israeli [21] and American cohorts [22]. These studies demonstrate the feasibility of ML models in gout prediction. With the proliferation of medical big data, the integration of ML with traditional statistical models will transform gout risk assessment from static prediction to dynamic etiological analysis.

**Fig. (1):**
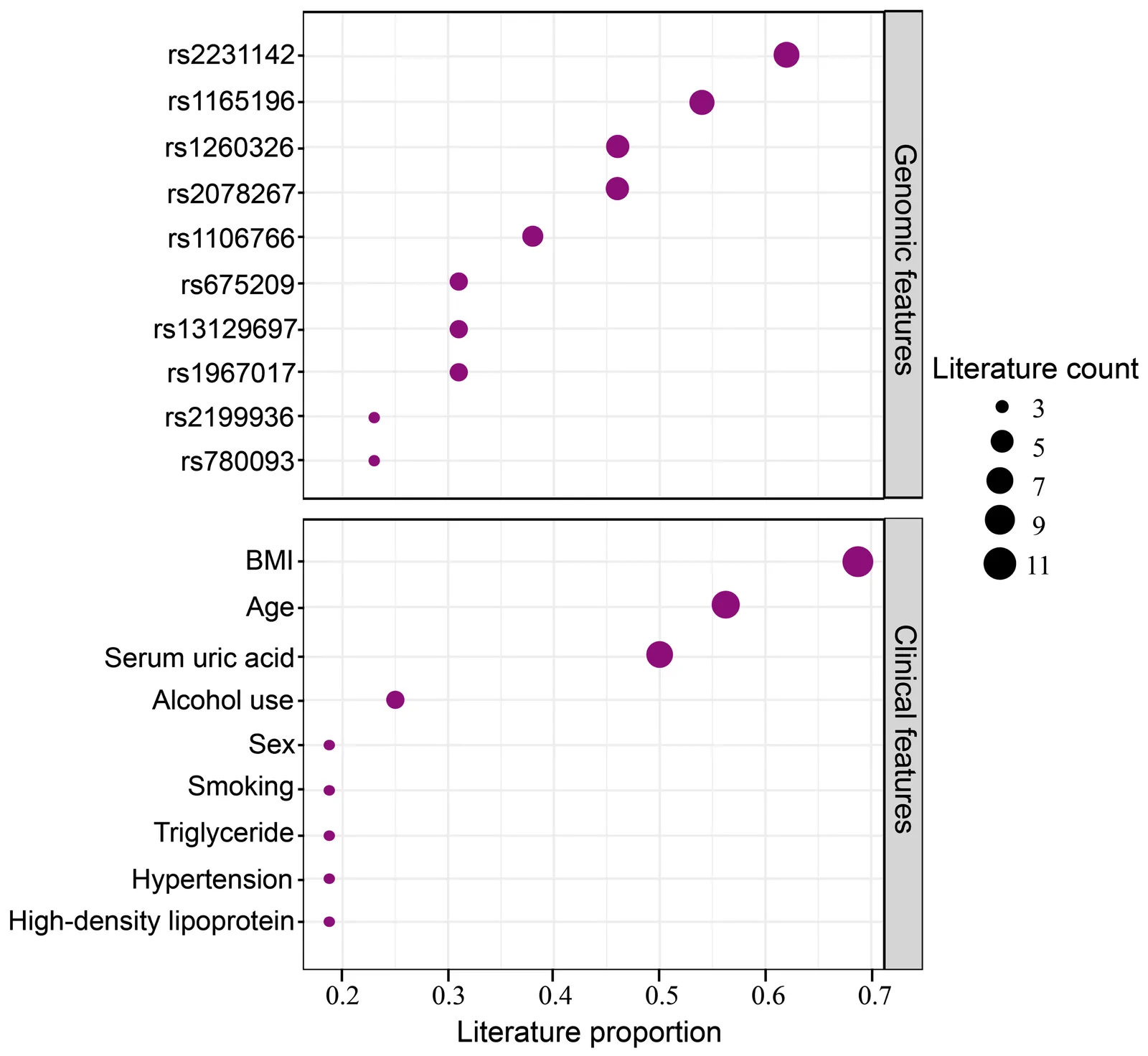
Robust prediction features of gout prediction models. These clinical and genomic features were selected by calculating their occurrence across the currently available 22 studies. The X-axis represents the proportion of literature, and the Y-axis represents genomic features (SNPs) and clinical features used in the model. The size of each dot represents the number of articles in which the feature appears.

In the feature selection processes of traditional statistical and machine learning prediction models, age, Body Mass Index (BMI), and serum uric acid levels have been consistently identified as gout predictors across multiple models (Fig. **1**). These three clinical features demonstrate significant predictive value for gout risk in diverse populations and cohorts worldwide, playing a crucial role in constructing gout risk prediction models. This consensus underscores the importance for clinicians to prioritize these features when assessing patients' gout risk [23, 24]. With advancements in multi-omics technologies, integration of multi-source data, and incorporation of multidimensional predictive biomarkers, the future will further enhance the early prediction capability and personalized guidance value of clinical feature-based models.

### 3.2 Gout Prediction Models Based on Genomics

PRS serves as a computational tool for quantifying individual disease susceptibility by aggregating risk effects from multiple genetic loci, typically derived from Genome-Wide Association Studies (GWAS) [25, 26]. PRS based on gout-associated single-nucleotide polymorphisms (SNPs) provides valuable support for gout risk prediction, enabling clinicians to develop personalized prevention and treatment strategies based on patients' genetic risk profiles to reduce the likelihood of gout onset (Table **3**).

Serum urate level is an independent gout risk factor, and genetic determinants of serum urate are closely linked to gout pathogenesis. In Caucasian populations, PRS constructed using urate- or gout-related SNPs demonstrated the ability to predict the risk of gout occurrence [27-30]. Similarly, studies in Japanese [31] and Chinese cohorts [32] utilized different SNPs from those in Western populations, but still showed good performance in identifying gout risk. The differences in SNP numbers and predictive performance among these population-specific models suggest that gout-associated SNPs may be subject to selection pressures, leading to varying predictive accuracy of PRS across ethnic groups. Therefore, developing universally applicable gout PRS for cross-population use will be a crucial direction for future gout prediction research [33-35].

In addition, disease-related SNPs identified through GWAS may confer susceptibility or protection. In a Taiwanese male cohort, Chang *et al*. [36] constructed separate PRS for risk and protective SNPs, which showed positive and negative associations with gout risk, respectively. These findings were further validated in a Taiwanese female cohort aged >50 years [37]. Thus, constructing PRS with SNPs that have consistent directional effects can avoid functional antagonism and improve prediction accuracy.

In SNP-based prediction models, ten SNPs including rs1260326 (*GCKR*), rs780093 (*GCKR*), rs2231142 (*ABCG2*), rs2199936 (*ABCG2*), rs1165196 (*SLC17A1*), rs1106766 (*R3HDM2*), rs13129697 (*SLC2A9*), rs2078267 (*SLC22A11*), rs675209 (*RREB1*), and rs1967017 (*PDZK1*) have been consistently identified as key components of PRS across diverse gout cohorts in multiple countries (Fig. **1**). These SNPs originate from genes involved, at their most basic level, in the transport and reabsorption of urate. They affect gout risk by perturbing urate transporters and disrupting urate homeostasis [38-40]. Thus, these results underscore the substantial influence of genetic factors on gout development and propose PRS as an advanced approach to applying genetic knowledge of gout in clinical practice.

### 3.3 Gout Prediction Models Based on Metabolomics

Gout is a metabolic disorder characterized by aberrant metabolite levels and dysregulated metabolic pathways [41]. As a method for qualitative and quantitative analysis of biofluid metabolites, metabolomics will be useful in constructing gout prediction models [42] (Table **2**). A study developed prediction models using urine-specific metabolites, achieving 100% recognition and 85% prediction efficacy for overall gout, with 83.3% and 89.9% accuracy for acute and chronic gout, respectively [43]. These results indicate that urinary metabolites not only exhibit excellent predictive capability but also hold potential for distinguishing disease states, facilitating the precision of clinical treatment strategy formulation [44-46].

Serological testing is an indispensable diagnostic method for gout management, as the dynamic equilibrium of uric acid and other metabolites in blood continuously influences gout pathogenesis. A logistic regression model incorporating three serum metabolites demonstrated exceptional diagnostic accuracy (AUC=0.956), indicating that serum metabolomic features are effective tools for early gout diagnosis [47]. Using multiple ML approaches, Shen *et al*. [48] not only constructed models to distinguish gout patients from healthy individuals but also developed a model to differentiate gout patients from hyperuricemia. Based on seven metabolites, these models achieved AUCs of 0.83-0.87 in the training set and 0.78-0.84 in the validation set. Collectively, these studies demonstrate the substantial potential of urinary and blood metabolites for gout prediction and diagnosis. Furthermore, metabolic profiles can serve as clinical markers to aid the precise screening of terpenes and terpene-based nanoformulations, thereby enhancing the therapeutic potential of these agents for alleviating gout-related inflammation [49].

### 3.4 Gout Prediction Models Based on Microbiomics

Microbiomics is all about understanding the makeup, roles, and organization of microbial communities in particular environments, along with how they interact with their hosts [50, 51]. In patients with gout, an imbalance in the gut microbiota can reduce levels of anti-inflammatory metabolites. This imbalance tends to increase the release of pro-inflammatory cytokines and can indirectly cause an excess of free radicals. Consequently, this exacerbates inflammation and speeds up the progression of the disease [52, 53]. By using 16S rRNA or metagenomic sequencing analysis, we can identify the specific microbiota linked to gout, which can help us build predictive models. This approach opens up new perspectives for more precise diagnosis and treatment of gout [54, 55] (Table **2**). Through 16s rRNA sequencing analysis, scientists identified five genera in the urinary microbiome of gout patients that showed significant changes. They developed a model that successfully distinguishes gout patients from healthy individuals, boasting AUC values of 0.971 for the test set and 0.934 for the validation set [56]. Metagenomics is an effective method for exploring the characteristics of microorganisms. Chen *et al*. [57] developed a Random Forest (RF) model that predicts gout with remarkable accuracy, boasting an AUC of 0.972. This model relies on 94 viral Operational Taxonomic Units (OTUs) linked to gout, which were identified through metagenomic data derived from virus-like particles. Similarly, RF has been used to identify three gut microbial genes specific to gout, achieving AUCs of 0.91 in the discovery cohorts and 0.80 in the validation cohorts, suggesting that these genes may serve as diagnostic markers for gout [58]. These studies show how microbiomics could help us predict gout. Changing gut microbiota and giving probiotics to those at high risk may prevent gout and help us personalize treatments.

## 4 GOUT PREDICTION MODELS WITH MULTI-SOURCE FEATURES

Gout, as a complex metabolic disorder, presents diagnostic challenges when depending solely on clinically based traditional models. Multi-omics approaches offer a transformative solution by providing multidimensional disease-related information, enabling a holistic understanding of disease pathogenesis and progression from divergent perspectives [59, 60]. By integrating omics risk factors with clinical traits, we can significantly improve disease prediction, laying a firmer foundation for early gout diagnosis.

### 4.1 Joint Prediction Models of Genomics with Clinical Features for Gout

Aside from genetic influences, your lifestyle and metabolic health are major factors that can lead to the onset of gout. Integrated predictive models incorporating genomic and clinical features offer new possibilities for gout risk prediction [61] (Table **3**). Several studies have validated that models combining PRS with lifestyle factors (including BMI, dietary patterns, sleep patterns, and alcohol intake) exhibit superior predictive performance compared to PRS alone in European American and Taiwanese cohorts [62-66]. In addition, the combination of PRS with demographic and metabolic characteristics in the European, American, and Asian cohorts has also improved the accuracy of gout prediction [67-70].

Furthermore, the integration of lifestyle factors, metabolic status, and genetic factors enables a more comprehensive assessment of gout risk. Liu *et al*. [71] developed highly accurate gout prediction models using the Taiwan Biobank dataset, incorporating 11 lifestyle and metabolic features along with the gout-associated rs2231142 genotype as predictive elements. The models demonstrated exceptional performance with random forest (AUC=0.986) and gradient boosting algorithms (AUC=0.987), illustrating that combining genetic susceptibility with lifestyle and metabolic characteristics maximizes gout prediction efficacy.

The integrated models demonstrate that, in addition to sex, lifestyle and metabolic traits, including alcohol consumption, smoking, triglycerides, high-density lipoprotein cholesterol, and hypertension, serve as significant clinical predictors across multiple models, indicating their close association with gout pathogenesis (Fig. **1**). Collectively, these clinical traits highlight various environmental influence factors on the disease progression of gout from different angles. They also show how we can increase the strength of risk SNP predictions by combining some of the synergistic effects of these genes. These studies suggest that integrating genetic data with many other factors could be a promising approach to improving the accuracy of gout prediction models. This achievement is important for real-time risk assessment and early management of patients.

### 4.2 Joint Prediction Models of Radiomics and Clinical Features for Gout

Radiomics, the high-throughput extraction and analysis of imaging features from medical images, has increasingly influenced disease diagnosis through advances in medical imaging and artificial intelligence [72]. There is solid evidence that models where radiomics integrates with clinical data can predict disease risk and thus enhance diagnostics [73, 74] (Table **3**). For example, a gout prediction model developed by integrating ultrasonic features with clinical characteristics identified four key predictors: serum uric acid, tophi, bone erosion, and double contour [75]. These predictors demonstrated optimal performance in a logistic regression model, with AUC values of 0.870 in the discovery set and 0.854 in the validation set. This study suggested that the combination of double contour with tophi and bone erosion enhances the diagnostic accuracy of ultrasound models in identifying gout [76].

These findings confirm that double contours, tophi, and bone erosion are effective imaging features for gout prediction [77, 78]. Meanwhile, prediction models integrating radiomics with clinical features show great potential in assisting physicians with the early diagnosis of gouty arthritis, reducing unnecessary joint puncture, evaluating the progression of gout, and holding promise to become important tools for precision diagnosis of gout.

## 5 PREDICTION MODEL OF GOUT-RELATED CLINICAL MANIFESTATIONS

Prediction models for pre-onset gout assist clinicians in appraising patients' gout susceptibility, permitting prompt diagnosis and intervention. Post-onset predictive models for various manifestations help further illuminate risk factors linked to disease progression, assess disease development trends, and direct treatment strategies [79-81].

### 5.1 Prediction Models for Distinguishing Gout from Similar Diseases

In the initial phases, individuals with gout may only display slight joint ache or fluctuations in blood uric acid levels. This occurs because their symptoms are frequently comparable to those of arthritis and other conditions, rendering prompt detection difficult. Developing models to detect gout early and separate it from comparable conditions should improve diagnostic accuracy. Metabolomics has been utilized for early gout detection, showing significant accuracy (AUC=0.829) in differentiating hyperuricemia from gout [82]. The merged model incorporating radiomics and multiple-source data has also been applied to identify gout patients among those with similar symptoms, reaching AUCs of 0.93 and 0.87 in the training and validation sets, respectively [83]. The ten features in this model, including clinical, laboratory, and imaging characteristics, may offer novel categorization standards for gout in the future. Furthermore, Lin *et al*. [84] merged clinical and radiomic features to build a diagnostic model for gouty arthritis that distinguishes it from rheumatoid arthritis and osteoarthritis (AUC=0.936), surpassing models relying solely on clinical or radiomic features.

These projects show how helpful it is to use information from all over to find diseases that act alike. But, there's not much work using other omics methods yet. For the future, studies could try to use all kinds of omics data to figure out gout.

### 5.2 Prediction Models of Post-Onset Management for Gout

The primary aim of post-gout management is to precisely evaluate the progression of the illness and subsequent hazards. Developing targeted prediction models offers a vital instrument for attaining this goal, primarily addressing urate crystal accumulation, frequent recurrence, and hospitalization events.

Monosodium urate (MSU) crystals are vital markers for assessing gout advancement and therapeutic effectiveness, guiding the modification of ULT based on MSU deposition hazards [85, 86]. Models founded on clinical features have shown excellent performance in MSU deposition prediction models. Researchers identified independent risk elements for MSU deposition, and the derived prediction model exhibited robust predictive capability (AUC=0.888) [87]. Further integration of clinical features with radiomics has exhibited greater responsiveness in detecting MSU deposition. In Chinese cohorts, predictive models combining clinical risk factors with radiomic characteristics from dual-energy CT (AUC=0.79) [88] or with musculoskeletal ultrasound features (C-index=0.774) [89] have shown good predictive capability. These studies suggest that multi-source feature merging models hold significant potential for constructing MSU deposition prediction models.

Gout is marked by high recurrence, with a minimum of two acute gout episodes within 12 months, clinically categorized as recurrent gout, generally indicating disease advancement. A multivariate logistic regression model built on risk factors from personal questionnaires and clinical examinations achieved an accuracy of 0.78 in predicting recurrent gout [90]. Omics data have also been employed to anticipate frequent gout. For instance, six different serum metabolites were utilized to construct an ML prediction model for frequent gout, with AUC values of 0.88 and 0.67 in the discovery and validation cohorts [91]. These models can effectively distinguish between frequent and non-frequent gout cases, and after clinical validation of their effectiveness, they are anticipated to enhance gout management and precision treatment planning.

Certain patients experiencing severe gout manifestations necessitate clinic stays and standard ULT, yet more than half of these individuals encounter gout outbreaks during the treatment timeframe. Consequently, identifying such high-risk persons is crucial for enhancing outcomes [92, 93]. Jatuworapruk *et al*. [94] identified nine clinical indicators of in-hospital gout in a New Zealand cohort, exhibiting peak predictive capability with C-statistics of 0.82 and 0.8 in the training and testing sets, respectively. This model was subsequently confirmed in an Asian group (AUC=0.71), indicating its flexibility across different demographics. The anti-clotting medication ticagrelor, utilized in people with acutely compromised heart arteries (ACS), may raise blood uric acid, possibly triggering gout. Investigators developed a logistic regression framework integrating beverage consumption, overall cholesterol, and initial serum urate levels to anticipate the hazard of gout during hospitalization for ACS patients on ticagrelor (AUC=0.784) and to assist practitioners in choosing alternative medications or starting rigorous oversight [95]. In summary, readily obtainable clinical features are useful for predicting gout episodes during a hospital stay and can guide detailed care plans for those most susceptible.

## 6 DISCUSSION

Gout is an inflammatory arthritis caused by urate crystal deposition, closely associated with hyperuricemia. The establishment of pre-onset and post-onset multidimensional prediction models helps identify high-risk populations, assists clinicians in promptly adjusting treatment strategies based on patients' risks of recurrence or tophus deposition, thereby facilitating precision treatment for patients.

Clinical features are widely used in gout prediction models for their cost-effectiveness, accessibility, and interpretability, enabling preliminary risk assessment but with accuracy potentially affected by confounders like medication use and comorbidities. The rapid advancement of multi-omics technologies has enabled their widespread application in full-course disease diagnosis and prediction, enhancing gout management precision. The PRS based on genomic variations facilitates clinical translation of gout genetic susceptibility with excellent predictive ability. However, PRS-based prediction has several limitations: cross-population applicability is restricted by genetic background differences(*e.g*., rs675209 and rs2199936 serve as predictive factors in Western cohorts, while rs1014290 is primarily associated with gout risk in Asian populations) [96], partial explanation of genetic variation, and neglect of gene-environment interactions [97, 98]. Metabolomic and microbiomic markers provide new targets for early prediction and diagnosis, yet their instability, environmental susceptibility, and inter-individual variability hinder model stability, reproducibility, and universal applicability. Radiomics aids gout prediction by detecting subtle lesions but is limited by variable imaging quality, high equipment requirements, and restricted rural hospital application, compromising its accuracy and clinical utility [99].

In light of the aforementioned challenges, substantial endeavors are still needed to advance the translational implementation of gout diagnostic and predictive models into routine clinical practice. The first step in the clinical transformation of a model is to conduct multi-center, cross-population external validation studies: it is necessary to integrate cohort data from different regions and races to verify the stability and accuracy of the model in a real clinical environment [100]. Meanwhile, for markers such as metabolomics and the microbiome that are susceptible to environmental interference, it is necessary to verify their predictive consistency among people with different dietary structures and lifestyles, and screen out core indicators with strong anti-interference ability and high universality. In the clinical transformation of the model, it's crucial to take cost-effectiveness into account [101]. A practical approach would be to emphasize low-cost, readily available conventional indicators for a basic model, saving multi-omics testing for the high-risk subgroups. This strategy helps keep costs in check while maintaining a solid balance between predictive performance and affordability.

## CONCLUSION

Models that predict gout before and after it starts are very important for handling the illness well. Since single technologies can not improve the precision of prediction models that use just one source of traits, studies show that multi-source prediction models, which mix multi-omics predictors with clinical traits, do better and add model precision. These models consider many aspects and include genetic and environmental factors in how gout occurs. This makes them good guides for deciding on care and advancing precision in medicine. The development of a universal gout prediction model faces notable clinical translational limitations, primarily stemming from the high costs associated with multi-omics detection technologies and the lack of sufficient multi-center, cross-population integrated datasets. These core bottlenecks hinder the standardization and popularization of such models in routine clinical practice and thus require targeted and in-depth resolution through subsequent research to advance their clinical translation and practical application.

## AUTHORS’ CONTRIBUTIONS

The authors confirm contribution to the paper as follows: study conception and design: H.Q, X.F; data collection: W.S; analysis and interpretation of results: W.S; draft manuscript: W.S, H.Q. All authors reviewed the results and approved the final version of the manuscript.

## Figures and Tables

**Table 1:** Summary of gout models across disease stages.

**Application Phase**	**Model Features**	**Model Type**	**Number of Studies**	**Sample Type**
Pre-onset phase	Clinical features	Single feature	3	Peripheral blood, serum and clinical data
	Genomics	Single feature	8	Peripheral blood
	Metabolomics	Single feature	3	Serum and urine
	Microbiome	Single feature	3	Stool and urine
	Genomics +Clinical features	Multi-source feature	11	Peripheral blood, serum and clinical data
	Radiomics +Clinical features	Multi-source feature	1	Serum and imaging
Post-onset phase	Clinical features	Single feature	4	Peripheral blood, serum, and clinical data
	Metabolomics	Single feature	2	Serum
	Radiomics +Clinical Features	Multi-source feature	4	Serum and peripheral blood clinical data and imaging

**Table 2:** Single-source features in gout prediction models.

**Model Features**	**Cohort**	**Features**	**Method**	**Predictive Ability (AUC)**	**References**
Clinical features	China	Serum creatinine, age, erythrocyte sedimentation rate, BMI, hemoglobin, white blood cell count, C-reactive protein, and serum uric acid	Logistic	0.953	[17]
	Israel	125 clinical features	XGBoost	0.781	[21]
		Serum uric acid, age, hyperlipidemia, non-steroidal anti-inflammatory drugs, and diuretic use	XGBoost	0.714	
	America	26 clinical features	LGBM	0.823	[22]
Metabolomics	China	Serum uric acid, hypoxanthine, xanthine, guanosine, inosine, and tryptophan	PLS-DA	Accuracy=85%	[43]
	China	Pyroglutamic acid, 2-methylbutyrylcarnitine, and L-phenylalanyl-L-phenylalanine	Logistic	0.956	[47]
	China	Glutamic acid, pyroglutamic acid, glycocholate, and lactic acid	RF, SVM, and Logistic	≈1	[48]
		Uracil, triglycyrrhizic acid, betaine, pentonic acid, myristic acid, arachidic acid ester, and glycocholate	RF, SVM, and Logistic	0.830-0.870	
Microbiome	China	Pandoraea, Campylobacter, Skermanella, Novosphingobium, and Sulfur bacterium	Logistic	0.971	[56]
	China	94 viral OTUs	RF	0.972	[57]
	China	3 microbial genes (Outer-α- sialidas, N-6 DNA methylase, and relaxase/mobilization nuclease domain protein)	RF	0.910	[58]

**Abbreviations:** XGBoost, extreme gradient boosting; LGBM, light gradient boosting machine; RF, random forest; GB, gradient boosting; PLS-DA, partial least squares discriminant analysis; SVM, support vector machine.

**Table 3:** Multi-source features-integrated gout prediction models.

**Model Features**	**Cohort**	**Multi-source Features**		**References**
Genomics +Clinical features	South Korea	30 gout-related SNPs	Lifestyle/metabolic state	[61]
	America	114 urate-related SNPs	BMI	[62]
	America	114 urate-related SNPs	Dietary	[63]
	Britain	30 gout-related SNPs	BMI	[64]
	Taiwan, China	3 gout-related SNPs	Alcohol use	[65]
	Britain	13 gout-related SNPs	Sleep pattern	[66]
	Japan	7 gout-related SNPs	Age, BMI, triglycerides, and glomerular filtration rate	[67]
	America	8 urate-related SNPs	Age, sex, type 2 diabetes, hypertension, BMI, and kidney disease	[68]
	Britain	114 urate-related SNPs	Age and sex	[69]
	Britain	17 gout-related SNPs	Age, BMI, and uric acid level	[70]
	Taiwan, China	rs2231142	11 Clinical features	[71]
Radiomics +Clinical features	China	3 radiomics features	Uric acid level	[75]

## LIST OF ABBREVIATIONS

ACS: Acute Coronary Syndrome
BMI: Body Mass Index
GWAS: Genome-wide Association Study
LGBM: Light Gradient Boosting Machine
ML: Machine Learning
MSU: Monosodium Urate
OTU: Operational Taxonomic Unit
PRS: Polygenic Risk Score
RF: Random Forest
SNP: Single-Nucleotide Polymorphism
ULT: Uric Acid-lowering Treatment
XGBoost: Extreme Gradient Boosting
